# CCT8 promotes cell migration and tumor metastasis in lung adenocarcinomas

**DOI:** 10.7150/jca.87983

**Published:** 2023-10-02

**Authors:** Zhiqiang Wu, Liyuan Deng, Jin Tao, Yuhai Lu, Xiaofei Zeng, Weikun Jia, Hu Chen

**Affiliations:** Department of Cardiothoracic Surgery, School of Clinical Medicine and The First Affiliated Hospital of Chengdu Medical College, Chengdu, 610500, China.

**Keywords:** LUAD, CCT8, AKT, Cell migration, Tumor metastasis

## Abstract

Chaperonins, which contain t-complex polypeptide 1 (CCT), are critical for correct protein folding to generate stable and functional protein conformations, which are important for cell growth and survival. However, little is known about the expression and prognostic significance of CCT8 (subunit 8 of the CCT complex chaperonin) in lung cancer. In this study, we demonstrated that CCT8 expression is frequently increased in human lung cancer. Survival analysis indicated that CCT8 expression is closely correlated with inferior overall survival in lung adenocarcinoma (LUAD), but not in lung squamous carcinoma (LUSC). Subsequently, ectopic expression of CCT8 facilitated cell migration and tumor metastasis, and vice versa. Mechanistically, CCT8 interacted and activated ATK. Inhibition of AKT suppressed CCT8-induced cell migration and tumor metastasis. Our findings support CCT8 as a biomarker for LUAD prognosis and as a target for LUAD therapy.

## Introduction

Lung cancer is one of the most common malignant tumors and the leading cause of cancer-related death worldwide 1. The high mortality is associated with a common diagnosis at an advanced stage, which hampers curative therapy. According to histology, lung cancer can be classified into two broad subtypes: small-cell lung cancer (SCLC), which is the cause of approximately 15% of cases, and non-small cell lung cancer (NSCLC), which accounts for the remaining 85%. In addition, NSCLC includes lung squamous carcinoma (LUSC), lung adenocarcinoma (LUAD) and large cell carcinoma subtypes 2. Rapid growth and proliferation of tumors is widely believed to be responsible for the high mortality and poor prognosis. Tumor cell rapid growth and proliferation cause a high demand for protein production. Newly synthesized proteins need to fold properly to exert their function. However, extrinsic factors (such as hypoxia, nutrient deprivation and acidosis) or intrinsic stresses (such as oncogenic activation, genomic instability and redox imbalance) result in the accumulation of unfolded or misfolded proteins at the ER lumen, which is known as ER stress 3. To adapt to ER stress, cells activate an adaptive mechanism to accelerate their protein folding capacity, termed the unfolded protein response (UPR) 4. Maladaptive UPR outputs trigger apoptosis 5. Our previous study found unresolved ER stress/UPR activates TAp73α to promotes colon cancer cell apoptosis 6. Therefore, targeting protein folding is an effective strategy for cancer therapy.

Besides UPR signaling, folding and quality control are crucial for the function of proteins when cells are in a normal condition 7. It has been reported that the cytoplasmic chaperonin containing TCP1 complex (CCT), also called TRiC (the TCP1 ring complex), plays an essential role in maintaining cellular homoeostasis by assisting the folding of many proteins 8. The CCT includes two identical stacked rings, each containing eight different proteins (CCT1-CCT8). CCT8 is a subunit of the CCT complex and is related to ATP enzyme activity 9. CCT8-controlled proteostasis is essential for extending *Caenorhabditis elegans* (*C. elegans*) lifespan by correcting proteostatic deficiencies in worm models of Huntington's disease 10, and is critical for T-cell maturation, selection, and function 11. In addition, many studies have shown that CCT, especially subunit 8 (CCT8), is highly expressed in B-cell non-Hodgkin's lymphoma 12, colorectal cancer 13, breast cancer 14, uterine sarcoma 15, glioma 16 and hepatocellular carcinoma 17. In addition, studies have also shown that CCT8 is overexpressed in colon cancer and hepatocellular carcinoma 8. However, the expression and prognostic significance of CCT8 in lung cancer is not clear.

The aims of the present study were to examine the expression of CCT8 and evaluate its prognostic significance in lung cancer. In this study, we demonstrated that CCT8 expression is frequently increased in human lung cancer. Survival analysis indicated that CCT8 expression is correlated closely with inferior overall survival in LUAD. CCT8 interacted and activated AKT to facilitate cell migration and tumor metastasis. These data suggest that CCT8 may be a biomarker for LUAD prognosis and a target for LUAD therapy.

## Materials and methods

### Cell culture

Human NSCLC cell lines (PC9 and NCI-H1299) and HEK-293T cells were purchased from the American Type Culture Collection (ATCC, Manassas, VA, USA). NSCLC cells were cultured in RPMI-1640 (Corning, Corning city, NY) supplemented with 10% heat-inactivated fetal bovine serum (FBS) (Gibco, NY) and 1% penicillin and streptomycin (P/S) (Gibco, NY). DMEM supplemented with 10% FBS was used to culture HEK-293T cells.

### Plasmid Construction and Lentivirus infection

For ectopic expression CCT8, human CCT8 gene was obtained by PCR then cloned into the pLVX-puro vector. For knockdown of CCT8 expression, pLentiCRISPR v2 plasmids carrying a single guide RNA (sgRNA) targeting a specific gene CCT8 (TGGTCCATATGCTGTACGAG) were purchased from Genscript (Piscataway, NJ). The recombinant plasmid, together with packaging (PsPAS2) and envelop plasmids (PMD2.G) (Addgene, Watertown, MA) were transfected into HEK-293T cells using PEI-max (Polysciences, PA, USA) to generate lentiviral particles. Virus was harvested after 72h post-transfection, filtered, and used for cell infection supplemented with 10μg/mL hexadimethrine bromide (polybrene; Sigma Aldrich, St. Louis, MO), Stable cell lines were generated by treating cells with puromycin (2 μg/ml; Gibco, New York, NY) for at least 2 weeks. An empty vector was used as a negative control.

### Colony formation assay

Colony formation assay was used to assess cell morphology. Cells were seeded at low confluence and grew for 14 days, then fixed with 4% paraformaldehyde and stained with 0.1% Crystal violet in 70% ethanol, and photographed by light microscope.

### Transwell assay

Transwell assay was used to measure the ability of cell migration according to our previous reports. Briefly, cells were suspended in serum-free media and seeded into the inner chamber (PC9, 5 × 10^4^ cells per chamber; H1299, 2 × 10^4^ cells per chamber). The outer chamber contained complete growth media. Cells were incubated for 12 h and then cells on the inside of the membrane were carefully removed with a cotton swab, while migrating cells on the outside of the membrane were fixed and stained with 0.5% Crystal violet in 70% ethanol, photographed under a light microscope. At least five random fields (200×) were photographed and cells were counted for each filed.

### Wound-healing assay

For wound-healing assay, cells were grown to 90% confluency in growth media, and then wounded with a plastic pipette tip. Cells were then washed twice with PBS and incubated in media containing 1% serum at 37 °C in a humidified incubator under 5% CO_2_. Cells were photographed under a light microscope (Nikon Eclipse Ti-S/L 100) after 12h of post-wounded.

### Immunoblot analysis and immunoprecipitation

For immunoblot analysis, cells were lysed in Cell lysis buffer (P0013, Beyotime, Shanghai, China) containing a protease and phosphatase inhibitor cocktail (P1048, Beyotime). Equal amounts of lysates were loaded, separated by SDS-PAGE, transferred to PVDF membrane (Millipore, Darmstadt, Germany). Membranes were blocked in 5% nonfat milk in TBST, probed with the indicated primary antibodies and HRP-conjugated secondary antibody for subsequent detection by enhanced chemiluminescence. anti-CCT8 antibody (67539-1-Ig, 1:1000) was purchased from Proteintech (Wuhan, China). Anti-AKT (total) (4821, 1:1000), anti-p-AKT(S473) (4060, 1:1000) antibodies were purchased from Cell Signaling Technology (Danvers, MA, USA). anti-E-cadherin (ab40772, 1:1000) antibody was purchased from Abcam (Cambridge, MA, USA). Anti-GAPDH antibody (AB0036, 1:5000) was purchased from Abways Technology (Shanghai, China).

For Co-immunoprecipitation experiments, PC9 cells and H1299 cells were lysed in lysis buffer (50 mM Tris HCl, pH 7.4, with 150 mM NaCl, 1 mM EDTA, and 1% Nonidet P-40) 18. Then the lysate was centrifuged at 15,000g at 4 ℃ to remove the precipitation. The supernatants were incubated with Rabbit-anti-CCT8 (67539-1-Ig, Proteintech, 1:100), or normal rabbit IgG (2729, CST, 1:100) on a rotator overnight at 4 ℃, followed by addition of protein A (sc-2001, Santa Cruz) agarose beads and incubation for a further 2h at 4 ℃. After four washes in PBST, samples were eluted in 2×sample buffer (125 mM Tris HCl, pH 6.8, with 4% SDS, 20% (v/v) glycerol and 0.004% bromphenol blue) to each sample for 10 min at 100 ℃, separated by SDS-PAGE and immunoblotted. Membrane was incubated with mouse-anti-AKT (4821, CST, 1:1000) and other primary antibodies as described in immunoblot analysis.

### Quantitative RT-PCR

Quantitative RT-PCR (qPCR) were used to detect the mRNA level and were performed as described previously 19. Briefly, total RNAs from cells were extracted using the TRIzol reagent (Invitrogen) according to the manufacturer's instruction. For first cDNA synthesis, total RNAs were performed reverse transcription using PrimeScript™ IV 1st strand cDNA Synthesis Mix (6215A, TakaRa, Japan). SYBR Green Master Mix reagent (Bio-Rad) and other reactants were carried out at 95 °C for 30 s, followed by 40 cycles of 95 °C for 5 s and 65 °C for 15 s in CFX96 Real-Time System (Bio-Rad). GAPDH was used as an endogenous reference gene to normalize target gene expression by the ΔΔCt method. QPCR primer sequences are listed below: GAPDH-F: 5'-TGGACTCCACGACGTACTCA-3', GAPDH-R: 5'-AATCCCATCACCATCTTCCA-3'; CDH1-F: 5′-GGATGTGCTGGATGTGAATG-3′, CDH1-R: 5′-CACATCAGACAGGATCAGCAGAA3′; CDH2-F: CTGACACTGGTGGCACTACTAA, CDH2-R: ATTCTGGTACACAATACAGAGGCA; MMP2-F: AGCTAATCAGCATTCTCACTCCTAC, MMP2-R: CACAGAAGGTTGTGAAAGGAGAAGA, MMP9-F: AGATTGGGAACCAGCTGTATTTGTT; MMP9-R: AAGAAGAAAAGCTTCTTGGAGAGCC; VEGFA-F: ATTGTGGAGGCAGAGAAAAGAGAAA, VEGFA-R: AACCGGTACAAATAAGAGAGCAAGA.

### In vivo metastasis assays

All animal experiments were performed in accordance with Guidelines for Animal Experiments at Chengdu Medical College with the approval of the Institutional Animal Care and Use Committee. Male athymic nude mice (4 weeks old) were purchased from GemPharmatech (Nanjing, China) and fed a standard chow diet. Mice were randomly assigned to three groups. PC9 stable cells were injected into the lateral tail vein of mice. One group of mice were injected with PC9 cells expressing empty vector (EV), two group of mice were injected with cells expressing CCT8. After 2 weeks of post-infection, mice injected with cells expression CCT8 were randomly assigned to treated and control groups (n = 4). Mice in the treated group were treated with MK2206 (120 mg/kg daily for two weeks) by gastric gavage, whereas the control group was treated with DMSO. Mice were monitored daily and sacrificed at 5 weeks post-infection. The lungs were dissected, fixed, and inspected for metastatic lesion, then subjected to H&E staining.

### Public database analysis

The “Gene_DE” module of the TIMER2.0 (the tumor immune estimation resource v2) web server (http://timer.comp-genomics.org/) was used to explore the differences between CCT8 mRNA levels in multiple tumor tissues and matched normal tissues on the base of the TCGA database.

The overall survival of lung cancer associated with CCT8 mRNA expression was analyzed in Kaplan-Meier Plotter (www.kmplotter.com) web server by using the dataset of “Lung cancer” of the “mRNA” group. The overall survival of LUAD and LUSC associated with CCT8 mRNA expression were analyzed by using the dataset of “Pan-cancer”, which derives from TCGA database, of the “mRNA RNA-seq” group. The overall survival of LUAD associated with mRNA expression of cell cycle-dependent proteins were analyzed by using the dataset of “Pan-cancer”, which derives from TCGA database, of the “mRNA RNA-seq” group.

### Immunohistochemistry using tissue microarrays

The immunohistochemistry of tissue microarrays (HLug-Ade150Sur-02-41799), including a total of 71 human lung adenocarcinoma, was purchased from Shanghai Outdo Biotech Company, Shanghai, China. Tissue microarrays were de-waxed in xylene, rehydrated through a graded series of ethanol (100%, 95%, 75%), rinsed in ultrapure water, and then immersed in 0.3% hydrogen peroxide (H_2_O_2_) for 30 min to block endogenous peroxidase. For antigen retrieval, sections were immersed in 0.01 mol/l of sodium citrate buffered saline (pH 6.0), boiled and maintained for 15 min in microwave oven. After being rinsed in PBS for 5 min, the slides were blocked with a solution of 3% bovine serum albumin (BSA) at room temperature for 60 min, and incubated at 4 ℃ overnight with the primary antibodies: anti-CCT8 antibody (1:1000 dilution; 67539-1-Ig, Proteintech, Wuhan, China). A subsequent reaction was initiated with a secondary antibody for 60 min at room temperature. The immunoreaction was visualized with diaminobenzidine (DBA) and counterstained with hemotoxylin, and then dehydrated and covered. To confirm the specificity of the immunostaining results, sections immunoreacted without the primary antibodies were used as negative controls.

To evaluate CCT8, expression, positively stained cells were counted. Counts were performed in high-magnification fields using the Automated Upright Microscope System (DM4000 B, Leica, Germany). The percentage of positive cells was determined by the three authors (Z. Wu, L. Deng, and J. Tao) independently. The intensity was scored as 0 (no immunostaining), 1 (weak immunostaining), 2 (moderate immunostaining) and 3 (strong immunostaining). The intensity of immunostaining score and the percentage of immunoreactive cells were multiplied to get IOD (integrated optical density) score. To compare the protein expression differences in the different specimen, the AOD (average optical density) score were calculate by using the formula: AOD=IOD/Area 20. The median value of the AOD were used as the cut-off values. Kaplan-Meier survival analysis was used to determine the relationship between CCT8 expression and patients' survival.

### Statistical analysis

Associations between variables were analyzed by performing chi-square tests for categorical variables and the Mann-Whitney *U* test for continuous variables. Overall survival (OS) was defined as the time from surgical resection to the date of death or last follow-up. OS were estimated using the Kaplan-Meier method, and nonparametric group comparisons were performed using log-rank tests. Comparisons between two groups of the experimental data were performed using the two-tailed unpaired Student's t-test. All statistical tests were two-sided, and the significance level was set at 5 %. Statistical analyses were performed using SPSS software for Windows version 16.0 (IBM Corporation, Armonk, NY, USA).

## Results

### CCT8 mRNA expression is associated with poor prognosis in LUAD

**Figure 1:**
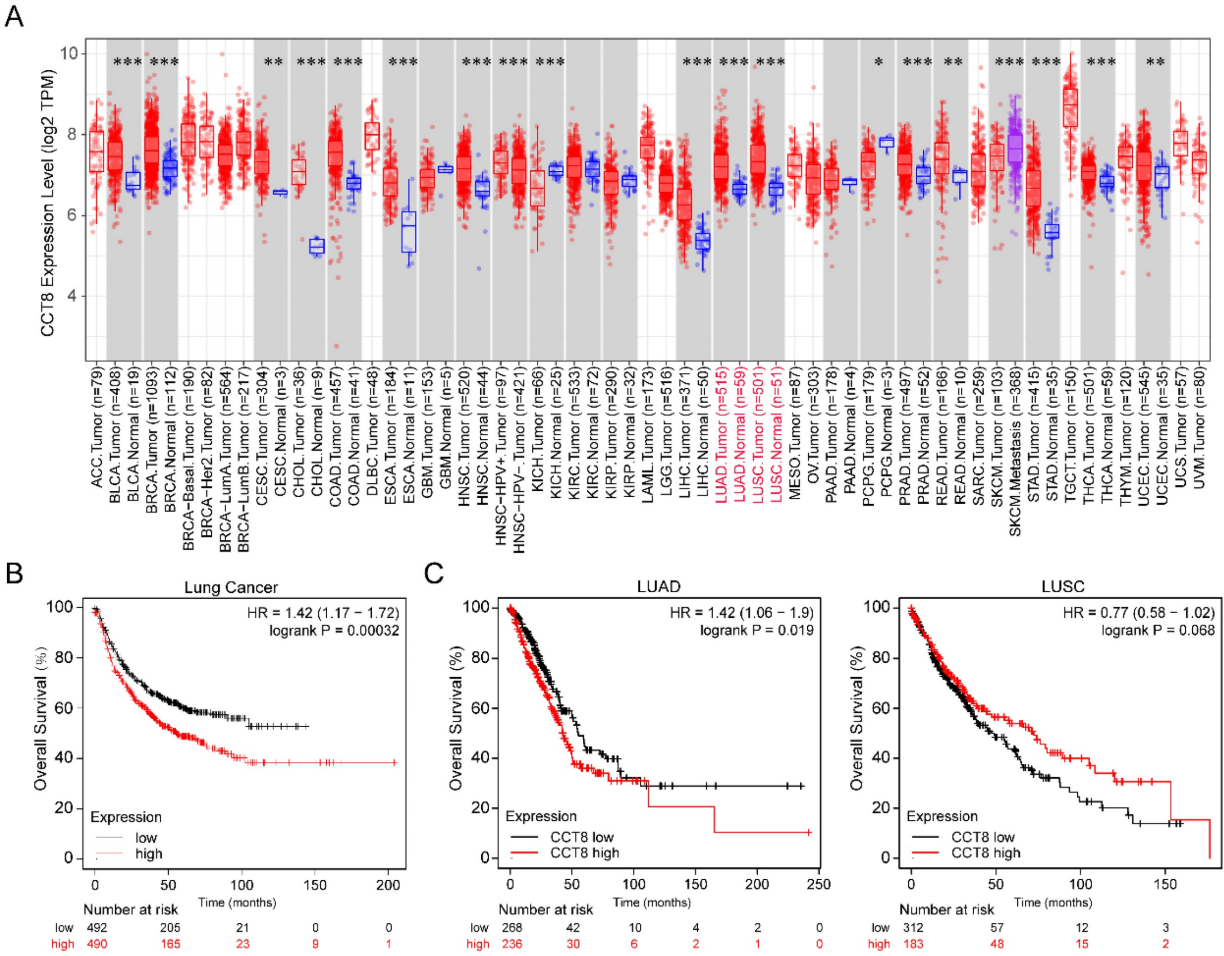
** CCT8 is highly expressed in lung adenocarcinoma cancer.** (A) The expression level of CCT8 in different cancers was analyzed using TIMER2.0, *p < 0.05; **p < 0.01; and ***p < 0.001. (B) Overall survival plots of lung cancer (n= 982) in Kaplan-Meier Plotter stratified by CCT8 mRNA expression. (C) Overall survival plots of LUAD (n = 504) and LUSC (n = 495) in TCGA stratified by CCT8 mRNA expression.

To investigate the expression level of CCT8 in tumor tissue compared with normal tissue, we analyzed publicly available datasets. In the TCGA database, CCT8 expression was significantly higher in some tumor tissues than in the matched control tissues, including bladder urothelial carcinoma (BLCA), breast invasive carcinoma (BRCA), cervical squamous cell carcinoma and endocervical adenocarcinoma (CESC), cholangiocarcinoma (CHOL), colon adenocarcinoma (COAD), esophageal carcinoma (ESCA), head and neck squamous cell carcinoma (HNSC), liver hepatocellular carcinoma (LIHC), lung adenocarcinoma (LUAD), lung squamous cell carcinoma (LUSC), prostate adenocarcinoma (PRAD), rectum adenocarcinoma (READ), stomach adenocarcinoma (STAD), thyroid carcinoma (THCA), uterine Corpus Endometrial Carcinoma (UCEC) (Fig. 1A, p < 0.001).

Since our research interest is lung cancer, we then investigated the association of CCT8 mRNA expression with the overall survival of lung cancer. As shown in Figure 1B, higher CCT8 mRNA expression was significantly associated with poor survival (p = 0.00032). Given that CCT8 mRNA expression increased obviously in both LUAD and LUSC (Fig. 1A), the main subtypes of lung cancer, we further analyzed the association of CCT8 expression with the outcome in LUAD and LUSC. As shown in Figure 1C, CCT8 expression in two subtypes of lung cancer results in different clinical outcomes; higher expression of CCT8 is associated with poor survival in LUAD patients, while higher expression of CCT8 is good for LUSC patients. These data indicate that CCT8 might be a diagnostic marker for the survival of LUAD patients.

### CCT8 protein expression correlated with poor prognosis in LUAD

To further confirm whether CCT8 expression was associated with overall survival in LUAD, we analyzed the CCT8 protein expression by using tissue microarrays. As shown in Table 1, the population of this tissue microarrays consisted of 38 men and 33 women, with a mean age of 60 years (range: 41-84 years). The tumor stage of the disease at pathological diagnosis was determined according to the UICC (The Union for International Cancer Control) guidelines of the TMN classification of malignant tumors and classified as follows: 11 in stage IA, 20 in stage IB, 16 in stage IIA, 4 in stage IIB, 10 in stage IIIA, and 10 in stage IIIB. The median follow-up period was 40 months (range: 1-58 months).

**Figure 2:**
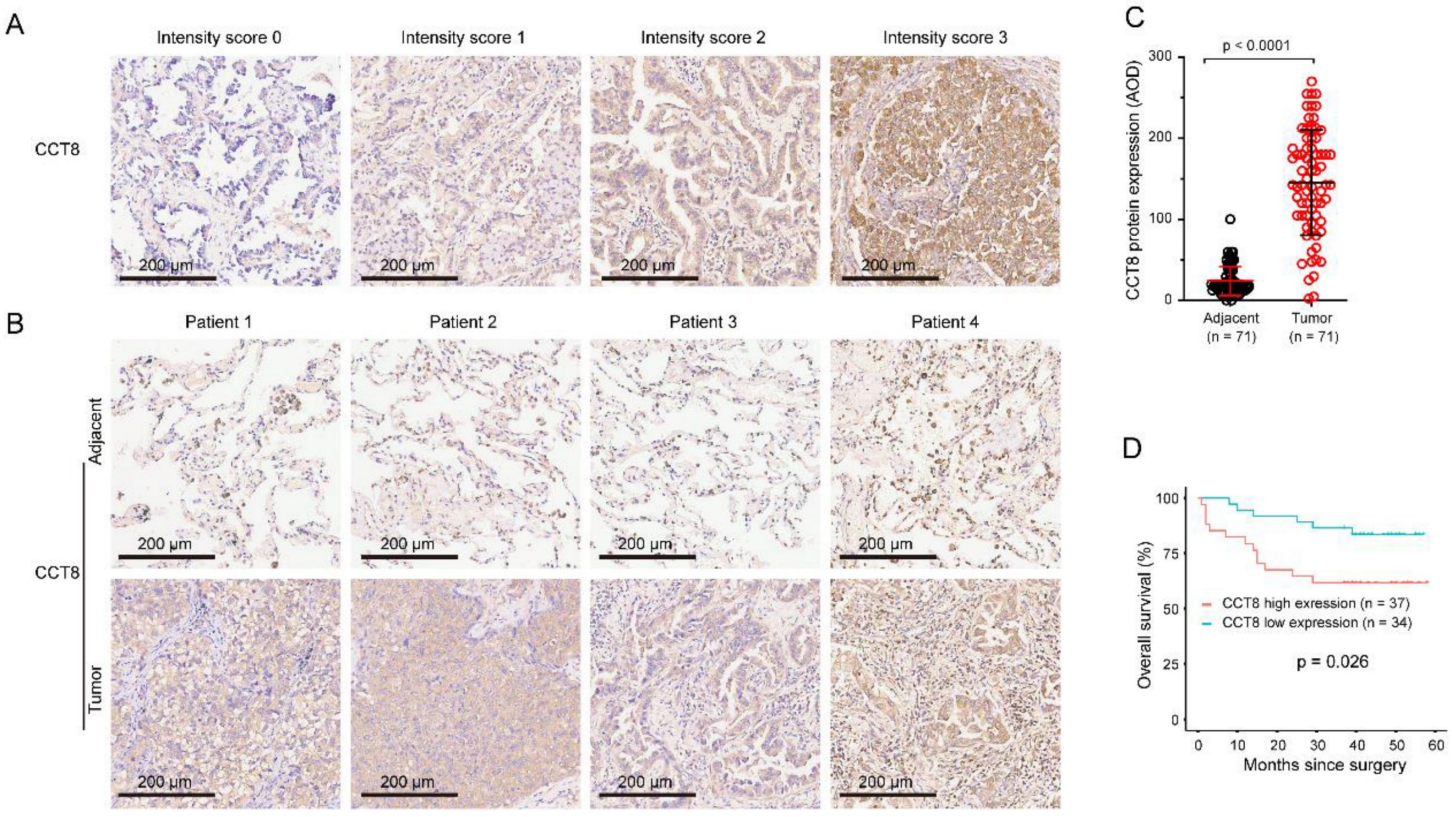
** Association between CCT8 protein expression and overall survival (OS).** (A) The intensity of staining was scored as follows: 0, no expression; 1, mild expression; 2, intermediate expression; and 3, strong expression. (B) Representative microphotographs of CCT8 immunoreactivities in lung adenocarcinoma and adjacent tissue. (C) Quantitative analysis of the expression of CCT8 in lung adenocarcinoma and adjacent tissue of the tissue microarray. (D) Association between OS and CCT8 protein expression. The OS of patients with high CCT8 expression was lower than that of patients with low CCT8 expression.

**Figure 3:**
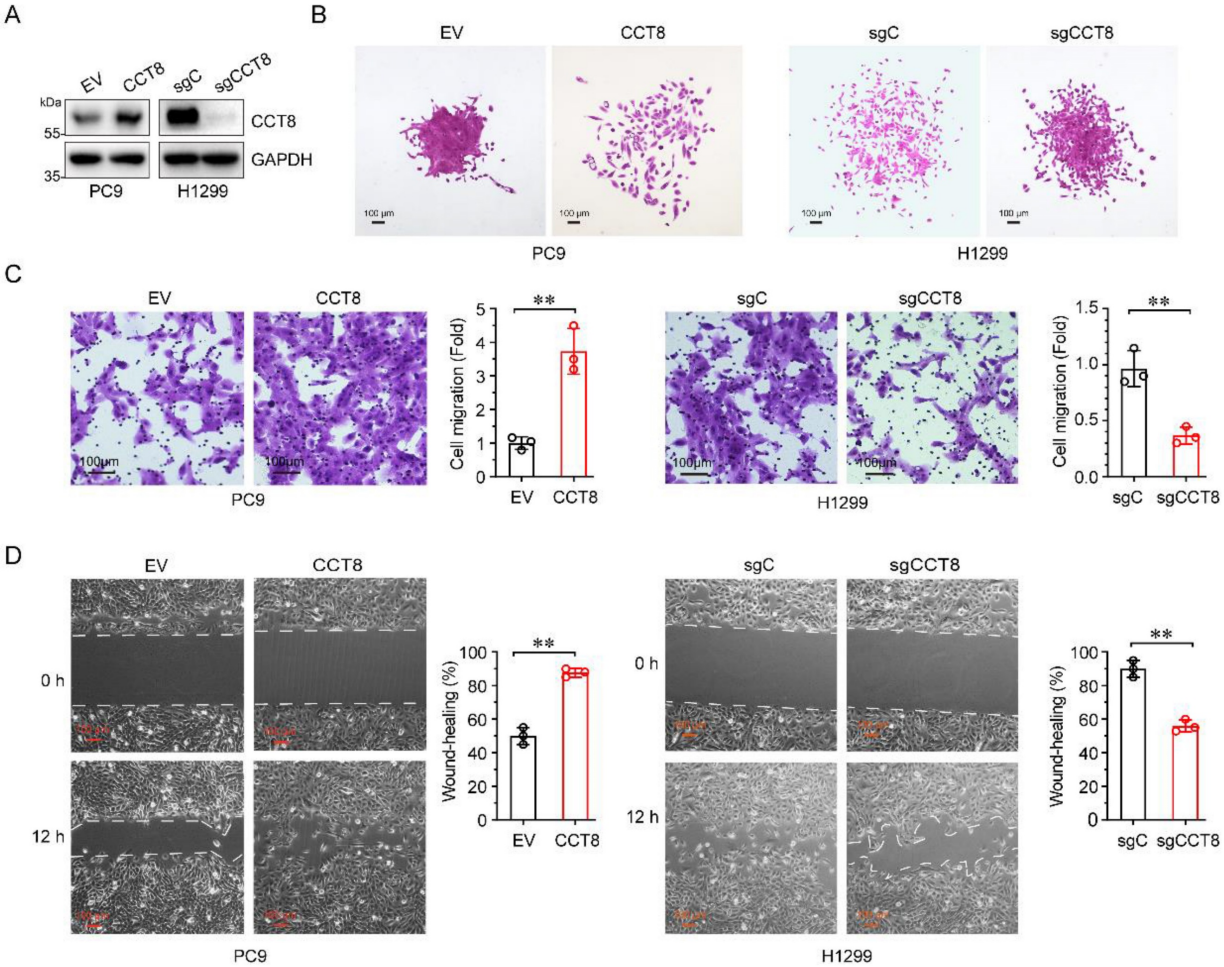
** CCT8 promotes cell migration.** Stable PC9 cells, which were infected with lentivirus encoding empty vector or CCT8, and stable H1299 cells, which were infected with lentivirus expressing sgC or sgCCT8, were subjected to immunoblot analysis for detection of CCT8 protein expression (A), colony formation (B), transwell assay (C), and wound-healing assay for testing of cell migration (D).

Specimens, paired LUAD and corresponding adjacent nontumor tissues, were stained by IHC. The staining intensity of CCT8 was scored as follows: 0, no expression; 1, mild expression; 2, intermediate expression; or 3, strong expression (Fig.2A). CCT8 was observed exclusively in the cytoplasm of tumor cells. As expected, CCT8 obviously increased in the tumor tissue of LUAD compared with its corresponding adjacent tissue (Fig.2B). Quantification analysis showed that CCT8 expression was significantly augmented in 71 LUAD tumor tissues compared with adjacent tissue (Fig.2C, p < 0.0001). Higher CCT8 protein expression was significantly associated with poor survival (Fig. 3D, p = 0.026). Taken together, these data further confirmed that CCT8 expression is increased in LUAD and that high expression of CCT8 is positively correlated with poor outcome.

We performed univariate analysis to determine the association between patient outcome (OS) and clinicopathologic factors or CCT8 expression. The results showed that CCT8 expression (p = 0.033), tumor size (p < 0.001), T stage (p = 0.021), N stage (p = 0.004), and TNM stage (p = 0.006) were significantly associated with poor OS. The OS of patients with high CCT8 expression tended to be worse than that of patients with low CCT8 expression.

### CCT8 promotes cell migration in LUAD

Over 90% of cancer-related deaths are caused by cancer metastasis 21. Given that high CCT8 expression in LUAD patients is associated with poor outcomes, we wanted to determine whether CCT8 promotes tumor metastasis. To explore the role of CCT8 in tumor metastasis, we constructed stable PC9 cells expressing CCT8 (Fig.3A) and examined the effect of CCT8 on cell mobility. As shown in Fig. 3B-3D, ectopic expression of CCT8 led to scattered cells growth (Fig. 3B), promoted cell migration (Fig. 3C), and increased wound-healing (Fig.3D). In contrast, knockdown of CCT8 by using CRISPR/Cas9 in H1299 cells resulted in an obvious change in cell morphology from spreading to compact colonies (Fig. 3B) and significantly suppressed cell migration (Fig. 3C) and cell mobility (Fig. 3D). Taken together, these data demonstrate that CCT8 plays an important role in cell migration.

### CCT8 promotes cell migration and tumor metastasis by activating AKT signaling

**Figure 4:**
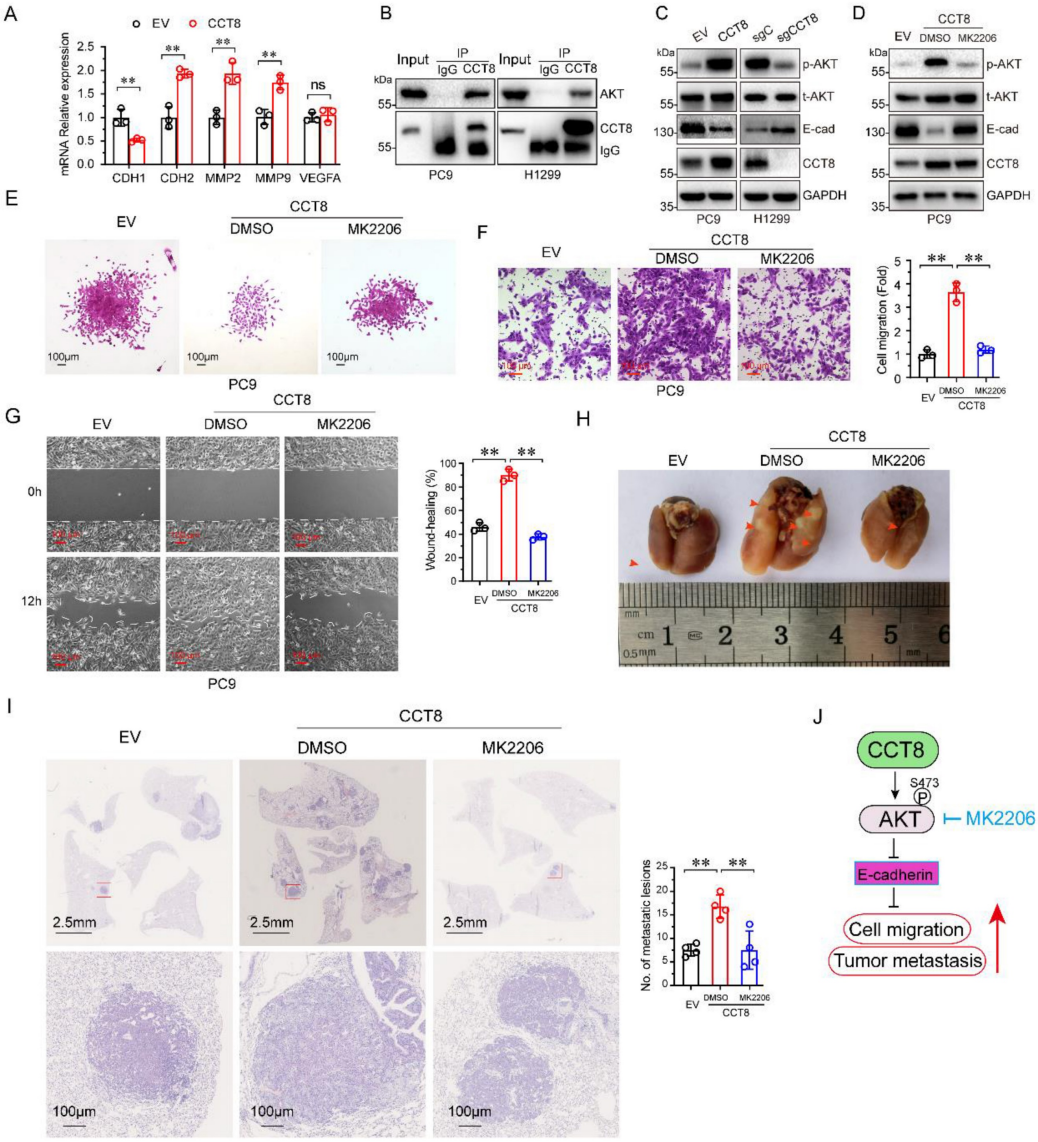
** CCT8 interacts with and activates AKT signaling to accelerate cell migration and tumor metastasis.** (A) PC9 stable cells, which were infected with lentivirus encoding empty vector or CCT8, were subjected to qPCR analysis for mRNA levels of CDH1, CDH2, MMP2, MMP9 and VEGFA. (B) PC9 cells and H1299 cells were subjected to anti-CCT8 (or anti-normal rabbit IgG) immunoprecipitation (IP), and coprecipitated endogenous AKT was detected by immunoblot analysis (IB). (C) PC9 stable cells, which were infected with lentivirus encoding empty vector or CCT8, and H1299 stable cells, which were infected with lentivirus expressing sgC or sgCCT8, were subjected to immunoblot analysis for protein levels of phosphorylated AKT (p-AKT), total AKT (t-AKT) and CCT8, Ecadherin (E-cad). (D-G) Stable PC9 cells were treated with MK2206 for 24 h and then subjected to immunoblot analysis for protein levels of phosphorylated AKT (p-AKT), total AKT (t-AKT) and CCT8, E-cadherin (E-cad) (D), colony formation (E), ranswell assay (F) and wound-healing assay (G). (H-I) Stable cells were injected into the tail vein of nude mice (4 mice per group). Two weeks after cell inoculation, these mice were treated with MK2206 (120 mg/kg daily for two weeks) or DMSO daily for two weeks. Lungs were dissected (H) and then subjected to H&E staining for histological analysis (left), and the lung lesions were counted (right) (I). (J) A working model showing that CCT8 activates AKT to promote tumor metastasis. **, P < 0.01; ns, not significant.

Metastasis is a dynamic and multi-step process, including in EMT, migration, invasion, angiogenesis, intravasation, extravasation, micrometastasis and macrometastasis 21. To investigate which process was affected by CCT8, we examined the expression of several genes which is important for tumor metastasis. As shown in Figure 4A, ectopic expression of CCT8 significantly downregulated the expression of CDH1 (encoding E-cadherin) concomitant with upregulated CDH2 (encoding N-Cadherin), MMP2, MMP9. However, VEGFA, which is critical to angiogenesis, had no change. Given that CHD1, CDH2, MMP2 and MMP9 play an important role in the process of EMT, we hypothesize that CCT8 mediate tumor metastasis through EMT. Our aforementioned data showed that CCT8 is mainly located in the cytoplasm by IHC staining, so we speculate that CCT8 may exert its function by interacting with other proteins. With this notion, we searched the interaction protein with CCT8 in BioGRID (http://thebiogrid.org), a database for protein-protein interactions, and found that CCT8 could interact with AKT, which is critical to tumor metastasis (Supplemental Table 1). We performed immunoprecipitation analysis in PC9 and H1299 cells, and found that CCT8 interacted with AKT (Fig. 4B). AKT functions mainly by its kinase activity. Ectopic expression of CCT8 significantly increased the level of phosphorylated AKT, the active form of AKT, concomitant with a decrease of E-cadherin expression, which is pivotal for cadherin junctions (Fig. 4C). As expected, knockdown of CCT8 obviously downregulated phosphorylated AKT, but upregulated the expression of E-cadherin (Fig. 4D). Blocking the activation of AKT by MK2206, a phase II inhibitor of AKT for breast cancer patients, rescued E-cadherin expression (Fig. 4D) and suppressed CCT8-induced cell-scattered growth (Fig. 4E), cell migration (Fig. 4F), cell mobility (Fig. 4G) and tumor metastasis (Fig. 4H-4I). These data demonstrate that CCT8 might activate AKT signaling to promote cell migration and tumor metastasis.

## Discussion

Chaperonin proteins are essential for cell survival and growth by helping proteins fold correctly and maintain stability and conformation. As an important subunit of the CCT complex, studies have shown that CCT8 is abnormally upregulated in many kinds of tumors and is correlated with the poor prognosis of tumor patients 22, 23. The detailed mechanism by which CCT8 promotes cancer progression remains to be elucidated. In this study, we symmetrically analyzed the mRNA expression of CCT8 by TIMER 2 on the basis of the TCGA dataset and found that CCT8 mRNA is higher in both LUAD and LUSC, the two main subtypes of lung cancer, than in normal specimens. Furthermore, we found that high expression of CCT8 correlated with shorter overall survival in LUAD, but not LUSC. Therefore, CCT8 might be used as a diagnostic biomarker for the prognosis of lung adenocarcinoma.

It has been reported that CCT8 can accelerate the G1/S transition 12 by regulating PCNA, CDK2 and Cyclin E to promote cell proliferation and tumor growth 13, 14, 16, 17. A recent study demonstrated that CCT8 could recover the wild-type p53-suppressed cell cycle 22. Metastasis is the worst form of cancer progression, and almost all cancer deaths result from metastatic disease 21. It has been reported that CCT8 can trigger colorectal cancer progression by enhancing the process of epithelial-mesenchymal transition (EMT) through upregulating snail, slug, twist, vimentin, which is suppressed by wild-type p53 22. However, the detailed mechanism of CCT8 -induced tumor metastasis is unknown. EMT, which is critical for tumor metastasis, is characterized by changes in cell morphology, as well as cell-cell and cell-matrix adhesions 24. Loss of E-cadherin, which often observed in metastatic tumors, results in cells to detach from their neighbors and begin to migrate 25. In this study, we found that overexpression of CCT8 could downregulate E-cadherin expression and trigger EMT, while knockdown of CCT8 can upregulate E-cadherin expression and inhibit EMT process. What's more, we identified that CCT8 could interact and activate AKT to promote cell migration and tumor metastasis. Our previous study demonstrated that blocking AKT with the small molecular inhibitor, MK2206, repressed mutant p53-induced cell migration 26. In this study, MK2206, an inhibitor of AKT, inhibited CCT8-induced cell migration and tumor metastasis, suggesting that CCT8 promotes cell migration and tumor metastasis by activating AKT (Fig. 4J). The mechanism of AKT activation by CCT8 needs to be elucidated further. We speculated that CCT8 may promote AKT binding to PDK1. In addition, how CCT8 expression is regulated is still unknown. In further work, we will study the upstream region of CCT8.

In general, this study has three prominent advantages. First, this study distinguished the prognostic role of CCT8 in LUAD and LUSC by using a public database. Second, a tissue microarray was used to confirm the results from public database. Third, we proved that CCT8 promoted cell migration and tumor metastasis *in vitro and in vivo*. However, several limitations should also be noted. First, there was a lack of CCT8 expression and an association with overall survival in LUSC. Second, the relationship between driver gene mutations (such as *EGFR* and Kras) and CCT8 expression in LUAD is unknown. Third, we used tissue microarray to verify the association of CCT8 and overall survival in LUAD; therefore, selection bias was inevitable during the selection of tumor areas for tissue microarray construction.

## Conclusions

In conclusion, we found that the expression of CCT8 is frequently increased in human lung cancer. Survival analysis indicated that CCT8 expression is closely correlated with inferior overall survival in LUAD. Ectopic expression of CCT8 facilitated cell migration and tumor metastasis, and vice versa. Mechanistically, CCT8 interacted and activated ATK. Inhibition of AKT suppressed CCT8-induced cell migration and tumor metastasis. All these points indicated that CCT8 may function as a therapeutic target for LUAD. Thereby, to clarify diagnostic value of CCT8 in LUAD pathogenesis, this early finding needs to be confirmed by studies with a larger group of patients.

## Supplementary Material

Supplementary tables.

## Figures and Tables

**Table 1:** Correlation between CCT8 expression and clinicopathological characteristics

	Variables	CCT8 expression		total	p value
		low	high		
Gender	male	20	18	38	0.236
	female	20	13	33	
Age (year)	≤60	19	16	35	0.15
	>60	21	15	36	
Grade	I/II	22	23	45	0.329
	III	17	9	26	
Tumor size	≤5.5 cm	33	25	58	**0.005**
	>5.5 cm	8	5	13	
T stage	T1/T2	30	26	56	1
	T3/T4	9	6	15	
N stage	N0	23	23	46	0.286
	N1-N3	16	9	25	
TNM stage	I	17	14	31	0.116
	II/III	22	18	40	

Significant p values are shown in bold.

**Table 2:** Univariate analyses of the factors correlated with overall survival of cancer patients

Variables	Univariate analysis		
	HR	95%CI	p value
CCT8 expression	2.869	1.089-7.558	**0.033**
Sex	0.606	0.238-1.539	0.292
Age	1.208	0.491-2.974	0.681
Grade stage	1.488	0.474-4.673	0.496
Tumor size	5.27	2.095-13.256	**<0.001**
T stage	3.008	1.183-7.653	**0.021**
N stage	3.999	1.574-10.161	**0.004**
TNM stage	7.984	1.811-35.197	**0.006**

Significant p values are shown in bold.
