# CCT8 promotes cell migration and tumor metastasis in lung adenocarcinomas: Erratum

**DOI:** 10.7150/jca.113359

**Published:** 2025-03-28

**Authors:** Zhiqiang Wu, Liyuan Deng, Jin Tao, Yuhai Lu, Xiaofei Zeng, Weikun Jia, Hu Chen

**Affiliations:** Department of Cardiothoracic Surgery, School of Clinical Medicine and The First Affiliated Hospital of Chengdu Medical College, Chengdu, 610500, China.

We recently found that there are two inadvertent mistakes in our article entitled “CCT8 promotes cell migration and tumor metastasis in lung adenocarcinomas”, due to our careless. Specifically,

- Figure 3D, the wound-healing images of sgCCT8 for the “0h” and “12h” group were un-intentionally misused during the image editing process. The correct images are provided below.
- Figure 4G, the wound-healing images were misused during the delivery from Microsoft PowerPoint to Adobe Illustrator. The correct images are provided below.

The correction will not affect the results and conclusion. The authors apologize for any inconvenience this may have caused.

## Figures and Tables

**Figure 3D:**
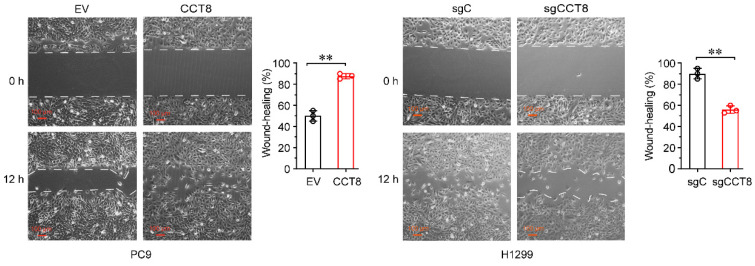
Stable PC9 cells, which were infected with lentivirus encoding empty vector (EV) or CCT8, and Stable H1299 cells, which were infected with lentivirus expressing sgC or sgCCT8, were subjected to wound-healing assay for testing of cell migration.

**Figure 4G:**
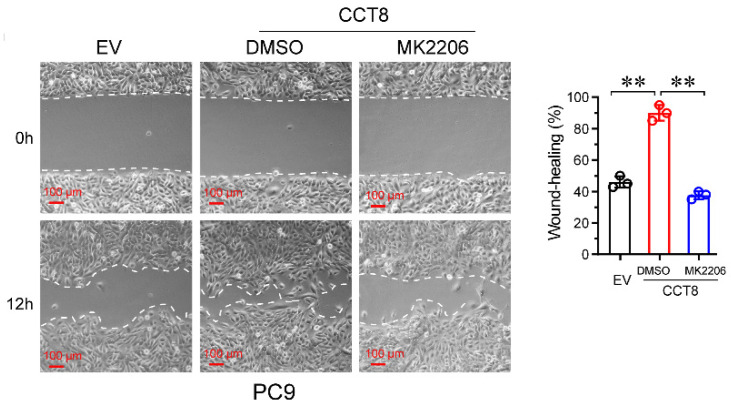
Stable PC9 cells, which were infected with lentivirus encoding empty vector (EV) or CCT8, were treated with MK2206 for 24 h and then subjected to wound-healing assay.

